# Supplementary material for: Improvement of the Chemical Reactivity of Michael Acceptor of Ethacrynic Acid Correlates with Antiproliferative Activities
Source: Molecules. 2023 Jan 16;28(2):910. doi: 10.3390/molecules28020910 (PMC9865193; doi:10.3390/molecules28020910)
Supplement: Supplementary file 1 [file molecules-28-00910-s001.zip › molecules-2049511-supplementary.pdf]

molecules

SUPPORTING INFORMATION

Improvement of the Chemical Reactivity of Michael Acceptor of Ethacrynic Acid Correlates with Antiproliferative Activities

Abdelmoula El Abbouchi[1,2], Nabil El Brahmi[1], Marie-Aude Hiebel[2], Hamza Ghammaz[3], Elmostafa El Fahime[3], Jérôme Bignon[4], Gérald Guillaumet[1,2,*], Franck Suzenet[2,*] and Saïd El Kazzouli[1,*]

1 Euromed Research Center, Euromed Faculty of Pharmacy, Euromed University of Fes (UEMF), Meknes Road, Fez 30000, Morocco
2 Institut de Chimie Organique et Analytique, Université d'Orléans, UMR CNRS 7311, BP 6759, CEDEX 2, 45067 Orléans, France
3 Centre National de la Recherche Scientifique et Technique (CNRST), Angle avenues des FAR et Allal El Fassi, Hay Ryad, 10102 Rabat, Morocco
4 Institut de Chimie des Substances Naturelles, CNRS, Université Paris-Saclay, Gif-sur-Yvette, France
* Correspondence: gerald.guillaumet@univ-orleans.fr (G.G.); franck.suzenet@univ-orleans.fr (F.S); s.elkazzouli@ueuromed.org (S.E.K)

Contents :

NMR (^{1}H and ^{13}C) for compounds **3-9**.. **S2-S8**

molecules

MDPI

The ^{1}H- and ^{13}C-NMR spectra of compounds **3-9**:

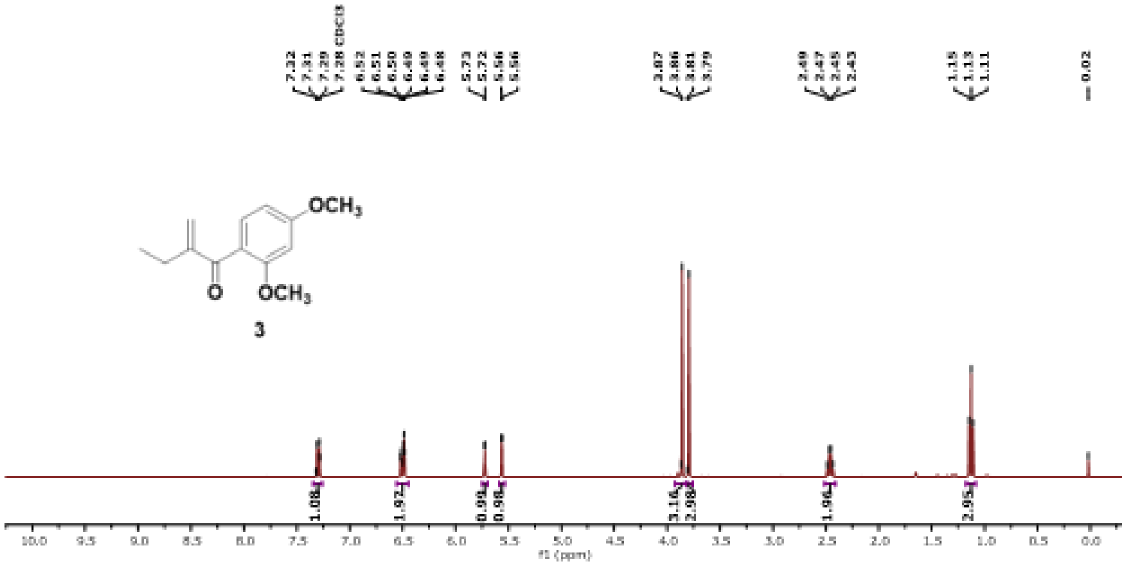

^{1}H NMR spectrum (400 MHz, $CDCl_3$) of compound **3**

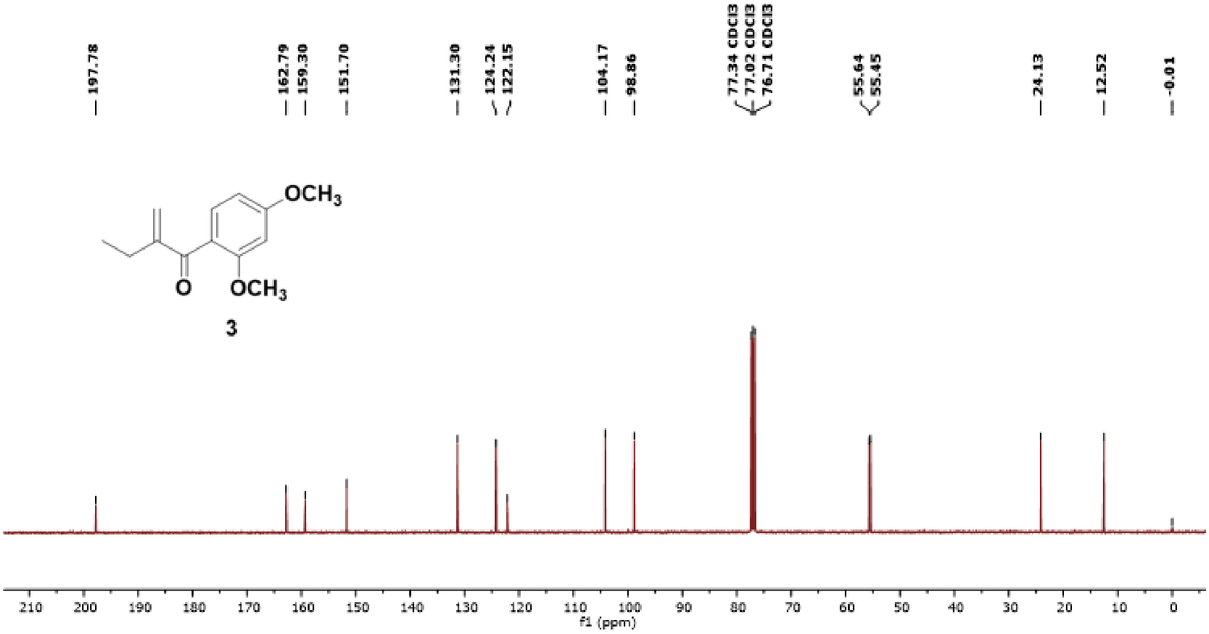

^{13}C NMR spectrum (101 MHz, $CDCl_3$) of compound **3**

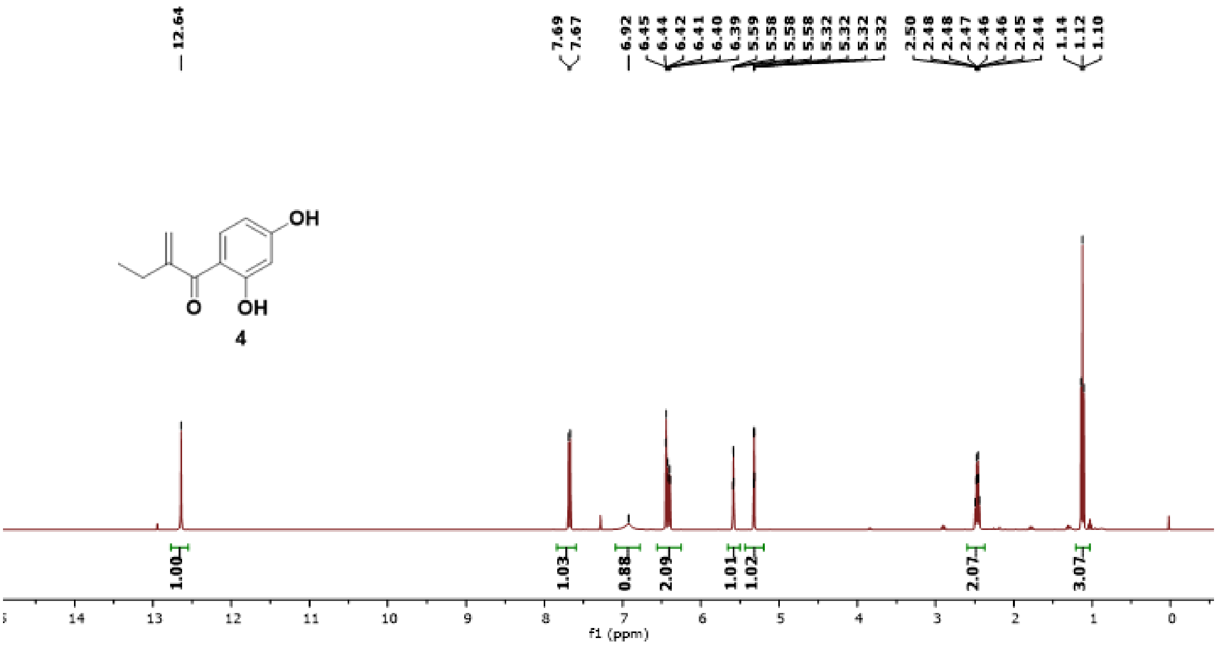

^{1}H NMR spectrum (400 MHz, $CDCl_3$) of compound **4**

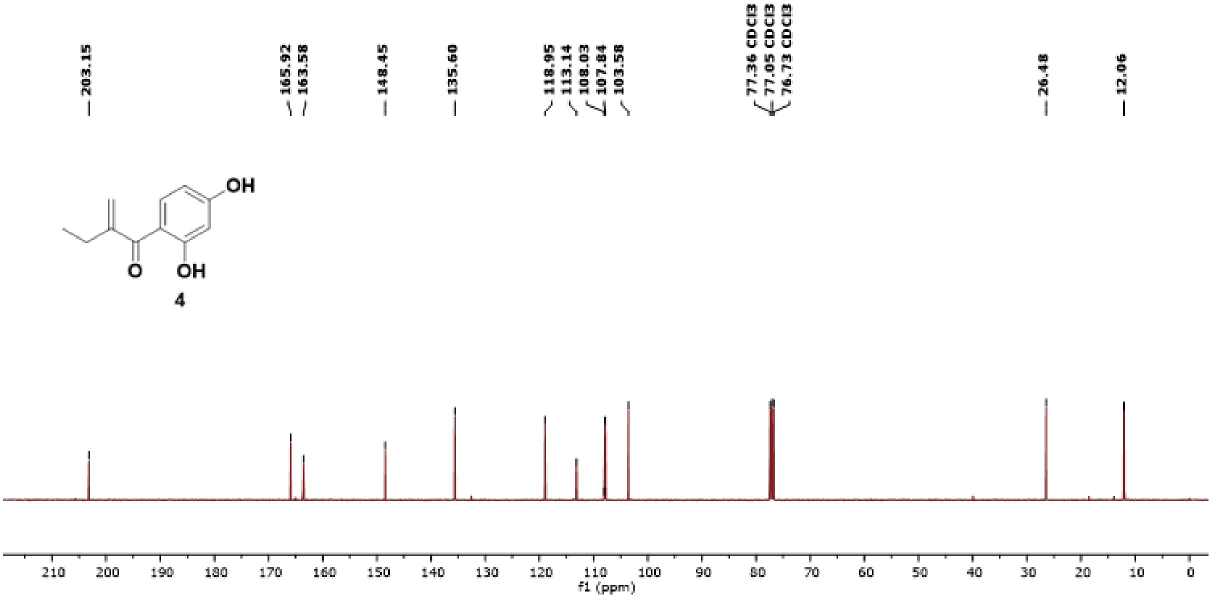

^{13}C NMR spectrum (101 MHz, $CDCl_3$) of compound **4**

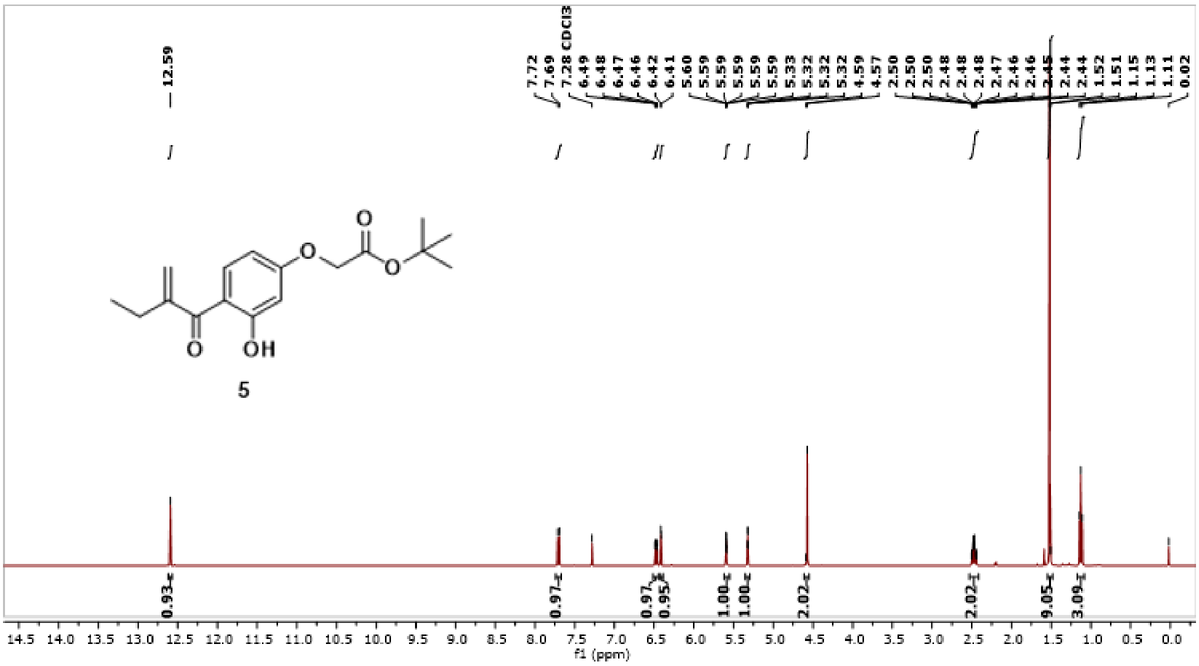

^{1}H NMR spectrum (400 MHz, $CDCl_3$) of compound **5**

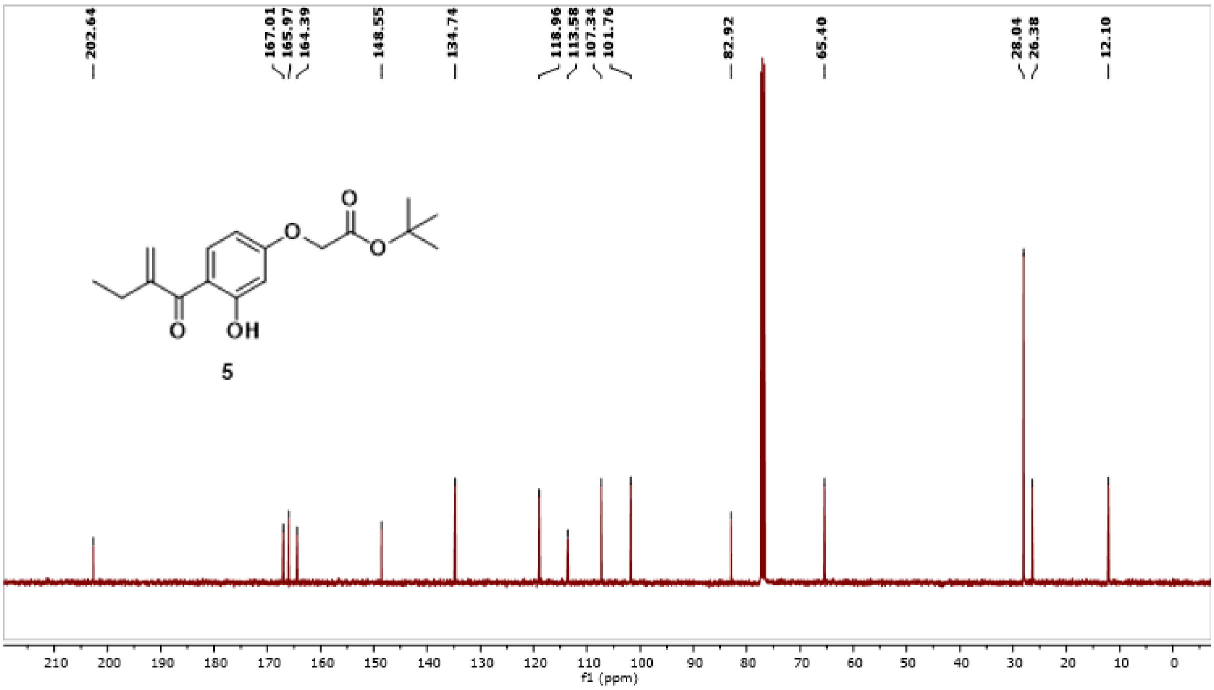

^{13}C NMR spectrum (101 MHz, $CDCl_3$) of compound **5**

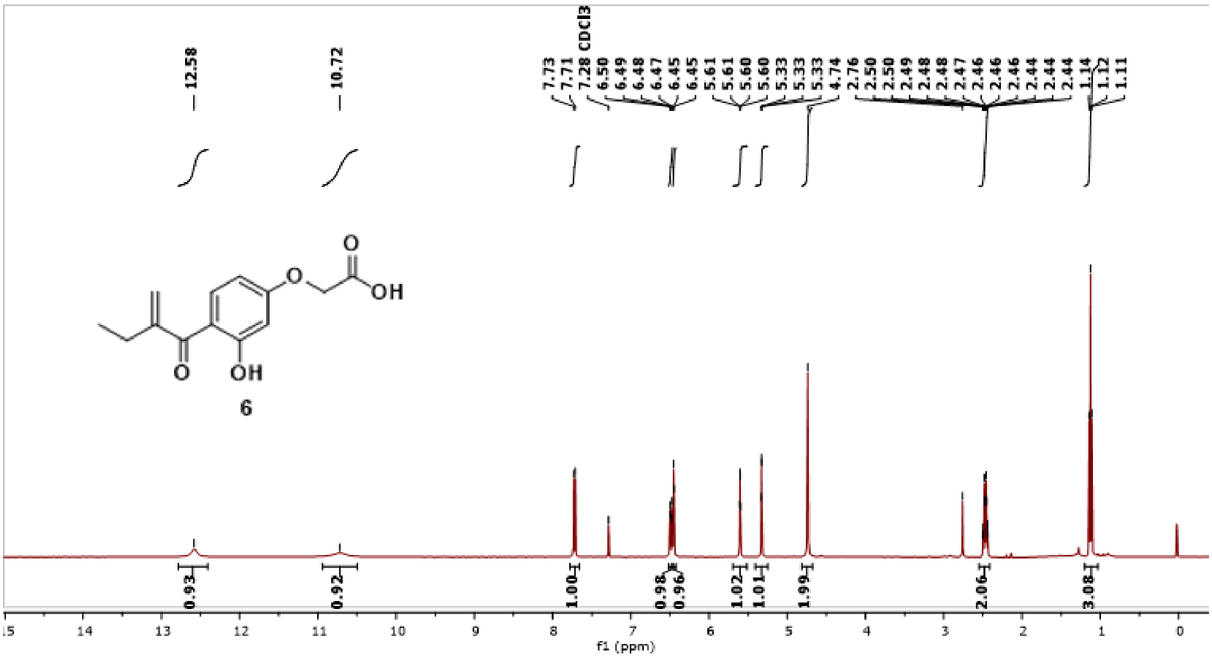

^{1}H NMR spectrum (400 MHz, $CDCl_3$) of compound **6**

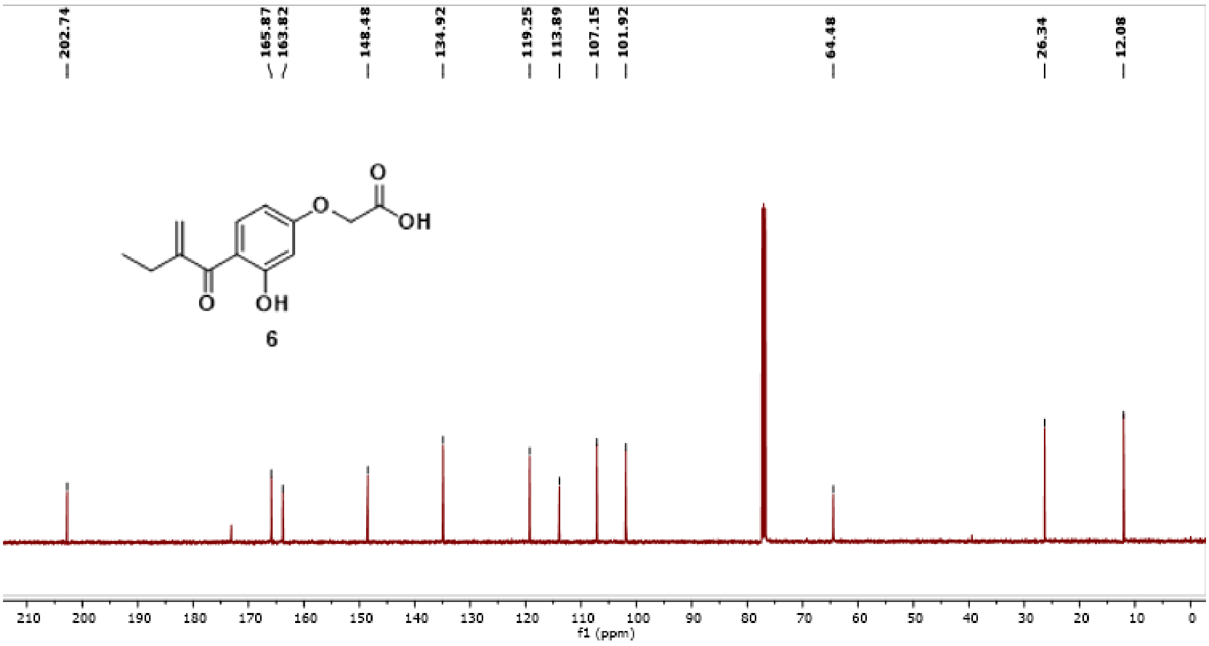

^{13}C NMR spectrum (101 MHz, $CDCl_3$) of compound **6**

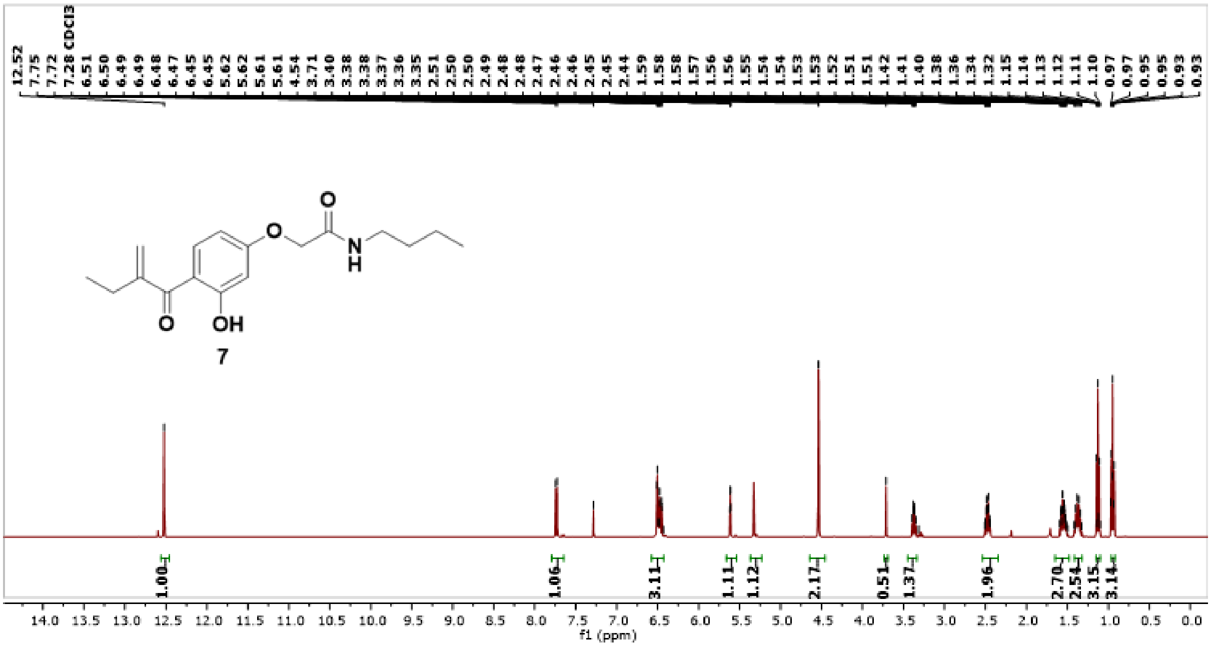

^{1}H NMR spectrum (400 MHz, $CDCl_3$) of compound **7**

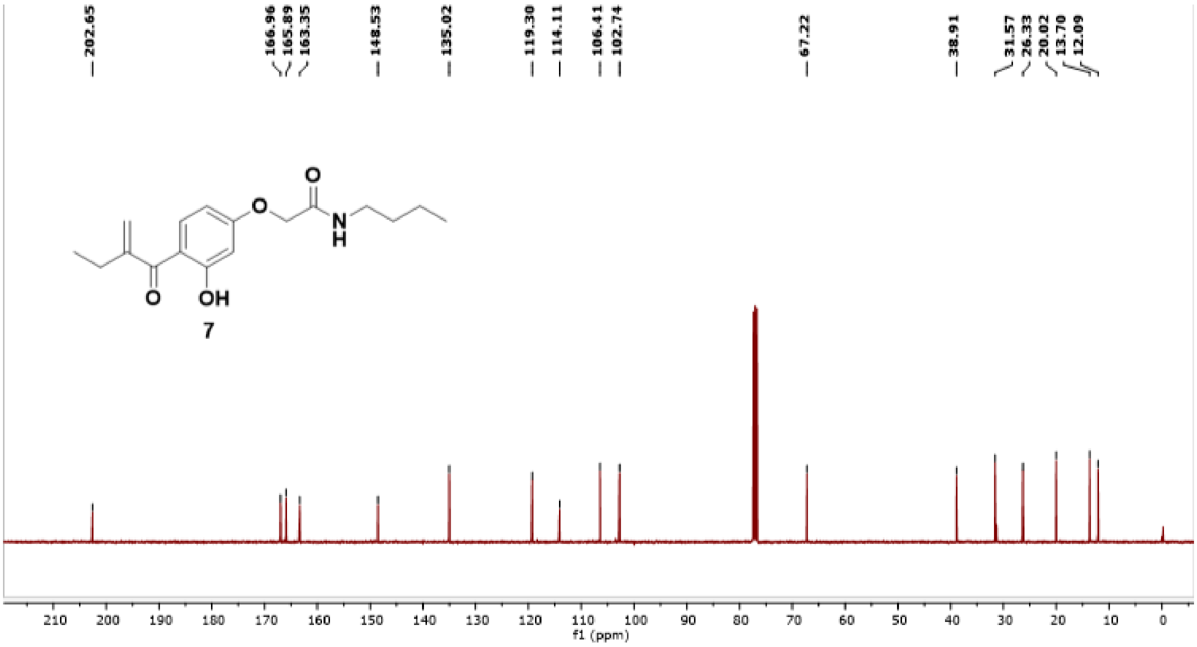

^{13}C NMR spectrum (101 MHz, $CDCl_3$) of compound **7**

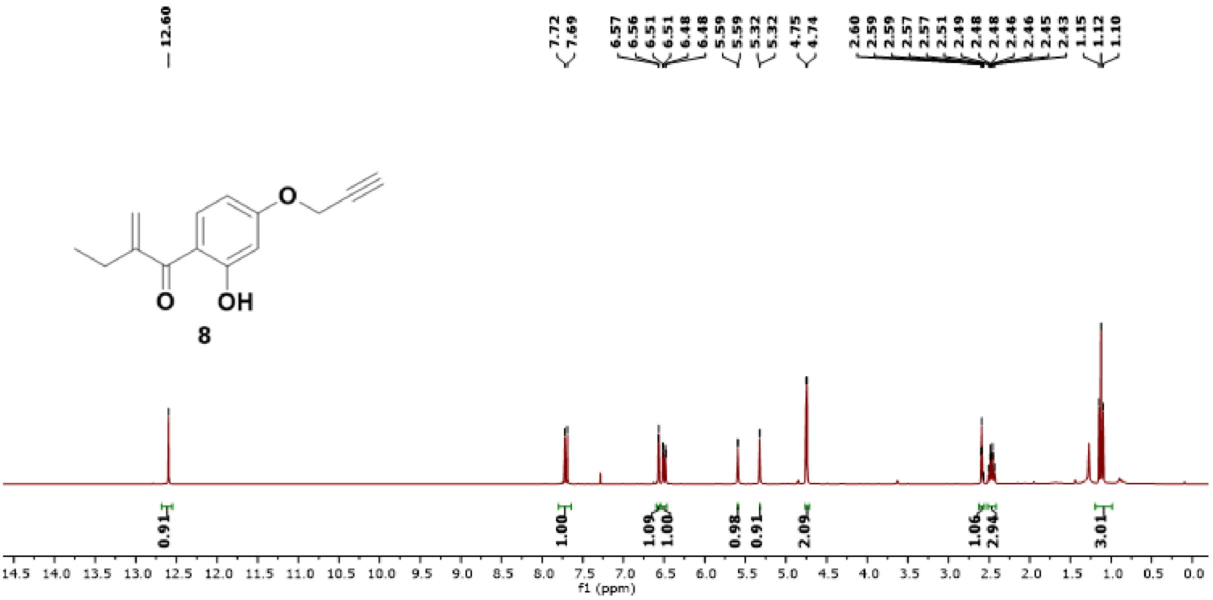

^{1}H NMR spectrum (300 MHz, $CDCl_3$) of compound **8**

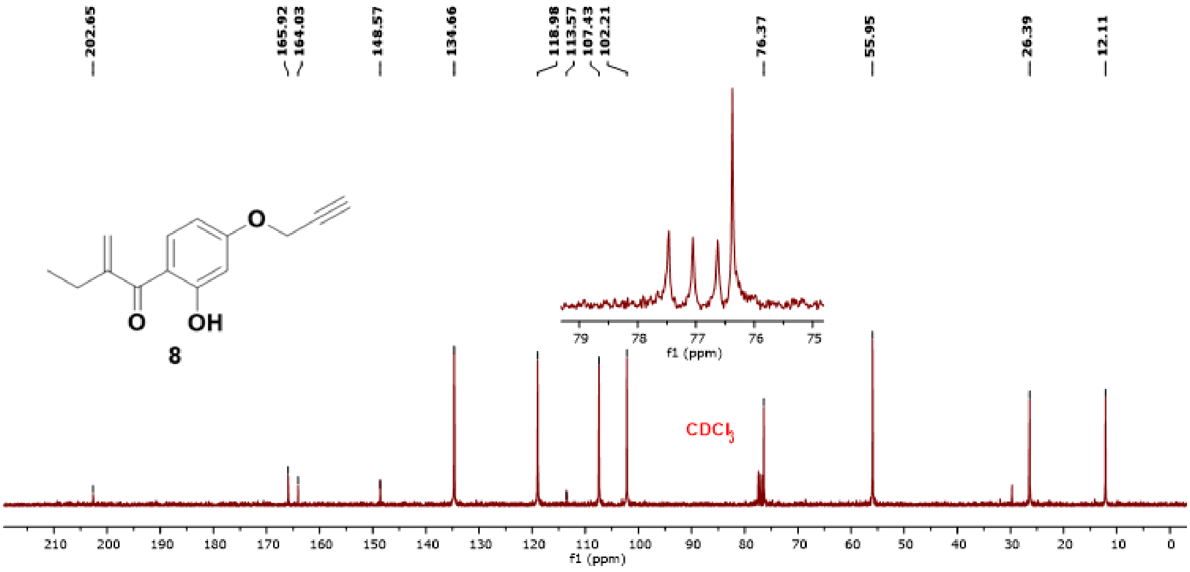

^{13}C NMR spectrum (75 MHz, $CDCl_3$) of compound **8**

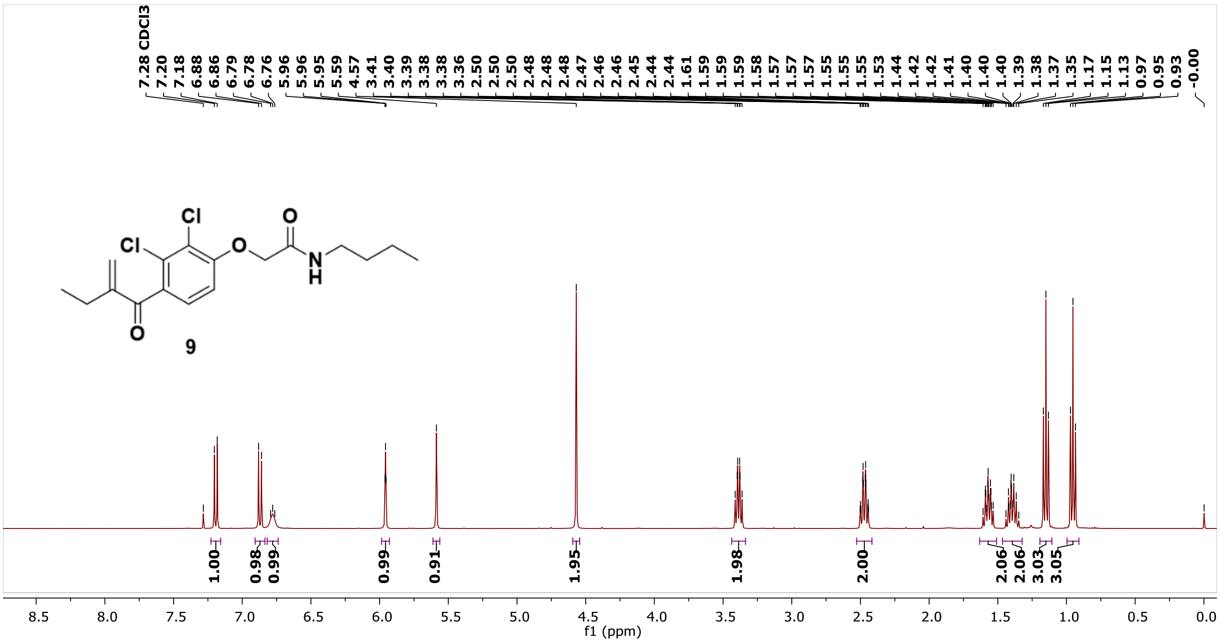

^{1}H NMR spectrum (400 MHz, $CDCl_3$) of compound **9**

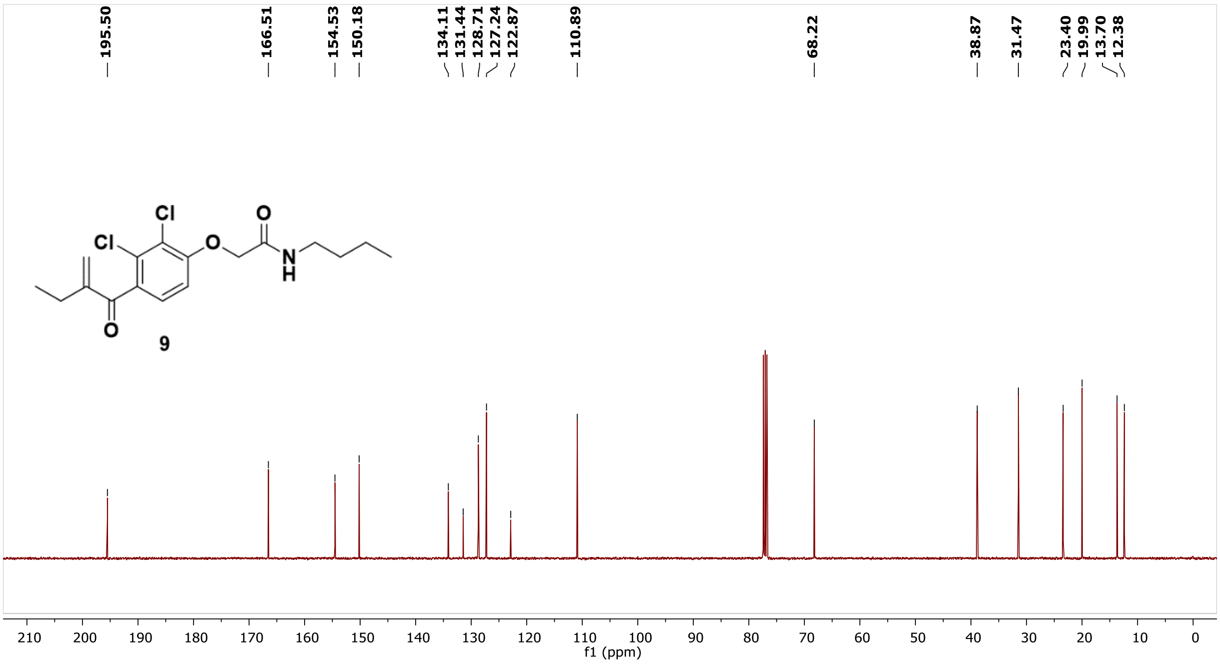

^{13}C NMR spectrum (101 MHz, $CDCl_3$) of compound **9**
